# Evaluation of changes in anthropometric indexes due to intermaxillary fixation following facial fractures

**DOI:** 10.15171/joddd.2016.039

**Published:** 2016-12-21

**Authors:** Javad Yazdani, Saeed Hajizadeh, Mohammad Ali Ghavimi, Bahram Pourghasem Gargari, Amin Nourizadeh, Yousef Kananizadeh

**Affiliations:** ^1^Department of Oral and Maxillofacial Surgery, Faculty of Dentistry, Tabriz University of Medical Sciences, Tabriz, Iran; ^2^Department of Oral and Maxillofacial Surgery, Faculty of Dentistry, Shahid Beheshti University of Medical Sciences, Tehran, Iran; ^3^Faculty of Health and Nutrition, Tabriz University of Medical Sciences, Tabriz, Iran; ^4^Department of Prosthodontics, Faculty of Dentistry, Tabriz University of Medical Sciences, Tabriz, Iran

**Keywords:** Anthropometry, body mass index, fixation, malnutrition, skinfold thickness

## Abstract

***Background.*** One of the treatment modalities for facial fractures is closed reduction technique, but treatment with intermaxillary fixation (IMF) interferes with normal nutrition, and malnutrition can affect the patient’s recovery. Anthropometric measurements such as skinfold thickness and body mass index (BMI) are universal indexes for diagnosing malnutrition. Therefore, in this study we explain how treatment with IMF changes the anthropometric indexes.

***Methods.*** In this study 60 patients were treated with 4 weeks of IMF. Skinfold thickness and BMI of these patients were measured and compared before and after the treatment.

***Results.*** Patients’ weight, BMI and skinfold thickness decreased during the IMF period, and this decrease was statistically significant (P < 0.01).

***Conclusion.*** Although no severe and acute malnutrition was seen among our patients, IMF led to mild to moderate malnutrition in some cases, making it necessary to use nutritional supplements.

## Introduction

Maxillofacial injuries commonly occur due to motor vehicle accidents, falls, insults and sport accidents.^1-3^ Mandible and midface fractures have high incidence in maxillofacial injuries and can be treated in two ways. The first treatment option includes open reduction techniques, which is carried out by surgical incisions and the fractured segments are fixed with different instruments like screws, plates and wires. The second option is closed reduction with the use of intermaxillary fixation (IMF), in which the fractured segments are immobilized adjacent to each other and this procedure results in the reunion of the separated segments.^4,5^ Despite the fact that IMF may result in some complications like malunion, nonunion, malnutrition and periodontal inflammation, it is used very widely when indicated.^5^ Duration of IMF depends on the type and location of the fracture, health condition of the patient and some other factors, but it is usually 2 to 6 weeks.^6,7^

During the IMF period the patient’s intake is affected because of the intermaxillary fixation.^8^ Many studies have shown a direct relation between nutrition and the healing process of the body;^8-13^ therefore, treatment with IMF could possibly effect the healing process. Some studies have demonstrated how IMF reduces body weight and other indexes like BMI.^14-17^ IMF is even used as a technique to treat extreme obesity.^18-21^ Malnutrition is associated with different signs and symptoms like losing more than 10% of body weight, neurologic changes, changes in skin, volume change of subcutaneous fat, hair loss, reduction of serum proteins and lipid factors, loss of muscle mass, etc.^21,22^ In this study we evaluated the effects of IMF on anthropometric measurements which are markers for malnutrition to show how treatment with closed reduction affects the patient’s nutrition so that we can think of solutions for this problem. Because nutritional habits are different in every region and society, it is necessary to evaluate the effects of closed reduction on people in each region to find the best way to face it.

## Methods

Based on the letter number 5/4/2013, this study was approved in 134th session of the Ethics Committee of the Tabriz University of Medical Sciences.

In this study, based on the results of other similar studies, 60 patients who were treated in the Oral and Maxillofacial Surgery Ward of Imam Reza Hospital, Tabriz, Iran, were analyzed. The patients aged 15 to 50 and had a BMI^14^ ranging from 18 to 30. For unifying the samples, only the patients who were to undergo 4 weeks of IMF were included. All the patients’ anthropometric indexes were assessed and recorded before treatment. Patients’ weight (Scale Weight Measuring Instrument, AWS Zeta Digital, USA) and height were measured, and BMI for each patient was calculated. Skinfold thickness^16^ was measured at three points; the triceps muscle (TSF, Triceps Skinfold), the biceps muscle of the arm and the submental region with a standard caliper (The Skyndex 1 SM 1000A Digital Skinfold Caliper, Wyoming, USA) by using a force of 10 gr/mm. The measurements are carried out ten times in each session and the mean values were documented. All the patients had a mandibular or midface fracture, necessitating closed reduction treatment with IMF. All the patients were treated with a 4-weeks period of IMF. None of the patients received any supplements during the treatment period. After the completion of the treatment the patients underwent all the anthropometric measurements again.

Statistical analyses were performed with SPSS 16.0 for Windows (SPSS INC., Chicago, USA). Quantitative data were presented as means ± standard deviations (SD), while qualitative data were demonstrated as frequencies and percentages (%). Data were analyzed with descriptive statistical methods and the mean difference t-test for independent groups. Statistical significance was set at P < 0.05.

## Results

Sixty patients were included in this study (36 males and 24 females) with n mean age of 28. The patients’ height ranged from 156 cm to 189 cm, with a mean of 172 cm. The patients’ weights were 49‒98 kg, with a mean of 69.45 kg before IMF. After 4 weeks the weight mean decreased approximately 2.64 kg to almost 66.81 kg (P = 0.025). The highest weight lost that was observed was 5 kg in one of the patients.

BMI average before the treatment among the patients was 23.11, which decreased 0.58, reaching 22.53 at the end of the treatment. These changes were statistically significant (P = 0.0341).

Average skinfold thickness at the biceps muscle area before treatment was 9.33 mm, with a range of 5.5‒20 mm, which decreased 0.54 mm during the treatment and reached 8.79 by the end of treatment (P = 0.009).

The skinfold thickness at the Triceps muscle decreased significantly (P = 0.011) (approximately 0.7 mm) during treatment. The mean changed from 12.04 mm to 11.33 mm. The skinfold thickness at the submental region decreased significantly (P = 0.007), too. The mean thickness at this region changed almost 0.58 mm, from 9.54 mm before the treatment to 8.96 mm after it.

According to classification of BMI,^22^ before treatment, 30% of patients were categorized as obese, 3 patients had mild malnutrition, one suffered from severe malnutrition and the rest had normal BMI. After the treatment period, 6 patients suffered from mild malnutrition.

TSF results showed that before the treatment only 2 patients had moderate malnutrition, but after the treatment 5 patients exhibited this condition.

## Discussion

In this study anthropometric measurements were obtained from patients and the results were analyzed. As mentioned previously, the patients in this study lost an average weight of 2.64 kg during 4 weeks of IMF, which was expected due to the lack of consumption of normal diet. Similar studies have confirmed these changes in weight because of IMF.^23^Worall showed a weight loss of 4.5 kg during 6 weeks of treatment;^24^in addition, Behbahani et al showed an average weight loss of 4.1 kg during 3.5 weeks.^25^ In a study in 2004, IMF was used as a treatment for patients who suffered from obesity; they lost an average weight of 7.4 kg using this technique.^26^ Although the weight loss showed in this study was less than that in other similar studies, it had the same pattern of reduction. It has been shown that a weight loss of more than 10% of the body weight is a sign of malnutrition.^27^Because weight loss did not reach 10% in this study, like most of the studies, we can assume that treatment with IMF did not result in obvious and severe malnutrition.

BMI was the other index which was evaluated in this study; it was shown that after 4 weeks of treatment with IMF, BMI had an average reduction of 0.58 units which was statistically significant. Two parameters affect BMI: patient’s height and patient’s weight. As the patient’s height remained the same during the study, with the reduction of the patient’s weight, BMI decreased, too. This has been confirmed in several other studies, too.^24,25^

The skinfold thickness depends on the amount of subcutaneous fat of the patient. This thickness differs in different points of the body; same body points are repeatedly used, such as triceps muscle of the arm, biceps muscle of the arm and submental region. As mentioned before, skinfold thicknesses in these 3 regions decreased significantly. Decreases in this index reflect the decrease in the amount of the subcutaneous fat in patients during the treatment due to malnutrition. The human body, in malnutrition conditions, uses the protein reserves first, and then the fat reserves are consumed. Therefore, the weight loss of patients in these conditions is usually due to the catabolism of muscles, and the reduction of the skinfold thickness is due to the consumption of body’s fat reserves. Therefore, it is reasonable to believe that 4 weeks of treatment with IMF can decrease skinfold thickness. Some studies have analyzed the skinfold thickness changes after IMF procedures and their results are consistent with those of the present study. In a study by Worall the skinfold thickness was measured at 4 points of the body and they showed decreases after IMF.^24^Antilla reported a decrease in skinfold thickness but the changes in their study were not statistically significant.^23^

Considering the normal ranges of BMI,^22,28^30% of patients participating in our study were overweight, 3 patients had mild malnutrition, one had severe malnutrition and the rest had BMIs in the normal range. After 4 weeks of IMF, patients with BMI over 18.5 changed from 97% to 93% and we had 6 patients with mild malnutrition and one with severe malnutrition. The normal ranges of TSF are shown in Table 1 when we began the study; according to our TSF measurements 9% of male patients and 5% of female patients were categorized as patients with mild malnutrition and 2 as patients with moderate malnutrition. At the end of the study, 12% of patients had mild malnutrition and 5 patients were categorized as patients with moderate malnutrition, indicating that treatment with 4weeks of IMF leads to patients’ mild and moderate malnutrition.

**Table 1:** The mean values of this study’s measurements

**Measurement**	**Before IMF (mean ± SD)**	**Before IMF (maximum)**	**Before IMF (minimum)**	**After IMF (mean ± SD)**	**After IMF** **(maximum)**	**After IMF** **(maximum)**	**P-value (mean)**
**Weight (kg)**	69.45(**±**1.6)	98	49	66.81(±1.4)	93	46	0.025
**BMI (kg/m** ^2)^	23.11(**±**0.83)	23.60	15.23	22.53(± 0.92)	30.40	14.97	0.0341
**Triceps skinfold (mm)**	12.04(**±** 0.51)	20	7	11.33(±0.62)	20	5.5	0.011
**Submental skinfold (mm)**	9.54(**±**0.32)	14	5.5	8.96(±0.68)	13	5	0.007
**Biceps skinfold (mm)**	9.33(**±**0.37)	20	5.5	8.79(±0.42)	19	5	0.009

In conclusion, we could show that with the local nutritional habits, based on patient’s weight and anthropometric indexes, treatment with IMF can result in malnutrition, though not severe. As mentioned before, good nutrition is the key for better and faster recovery; therefore, when closed reduction techniques are used for treatment in the maxillofacial region, a supplemental nourishment planning is mandatory.

Since nutritional habits are different in different regions we recommend that similar studies be carried out in different regions with different nutritional habits and with the use of different supplements to find out the best nutritional plan in each region to avoid malnutrition in patients.

## Acknowledgments

The financial support of the Tabriz University of Medical Sciences is acknowledged.

## Authors’ contributions

All the authors participated in carrying out this study and supervised writing this article. JY, SH, MAG, BPG, and AN designed the study. SH and YK performed the literature review and searched in databases. Furthermore, JY, SH, MAG, and YK performed data analysis. All the authors have read and approved the final manuscript.

## Funding

This study was funded by Tabriz University of Medical Sciences.

## Competing interests

The authors declare no competing interests with regards to the authorship and/or publication of this article.

## Ethics approval

Considering the letter number 5/4/2013, this study was approved in 134th session of the Ethics Committee of Tabriz University of Medical Sciences.
